# Aberration correction in diagnostic ultrasound: A review of the prior field and current directions

**DOI:** 10.1016/j.zemedi.2023.01.003

**Published:** 2023-02-26

**Authors:** Rehman Ali, Thurston Brevett, Louise Zhuang, Hanna Bendjador, Anthony S. Podkowa, Scott S. Hsieh, Walter Simson, Sergio J. Sanabria, Carl D. Herickhoff, Jeremy J. Dahl

**Affiliations:** a Department of Imaging Sciences, University of Rochester Medical Center, Rochester, NY, USA; b Department of Electrical Engineering, Stanford University, Stanford, CA, USA; c Department of Radiology, Stanford University School of Medicine, Stanford, CA, USA; d Department of Radiology, Mayo Clinic, Rochester, MN, USA; e University of Deusto/ Ikerbasque Basque Foundation for Science, Bilbao, Spain; f Department of Biomedical Engineering, University of Memphis, TN, USA

**Keywords:** Ultrasound, Beamforming, Aberration, Aberration correction, Phase aberration, Speed of sound

## Abstract

Medical ultrasound images are reconstructed with simplifying assumptions on wave propagation, with one of the most prominent assumptions being that the imaging medium is composed of a constant sound speed. When the assumption of a constant sound speed are violated, which is true in most in vivo or clinical imaging scenarios, distortion of the transmitted and received ultrasound wavefronts appear and degrade the image quality. This distortion is known as aberration, and the techniques used to correct for the distortion are known as aberration correction techniques. Several models have been proposed to understand and correct for aberration. In this review paper, aberration and aberration correction are explored from the early models and correction techniques, including the near-field phase screen model and its associated correction techniques such as nearest-neighbor cross-correlation, to more recent models and correction techniques that incorporate spatially varying aberration and diffractive effects, such as models and techniques that rely on the estimation of the sound speed distribution in the imaging medium. In addition to historical models, future directions of ultrasound aberration correction are proposed.

## 1 Introduction

As early as the late 1960’s, researchers in the field of diagnostic ultrasound identified broadening and distortion of the ultrasonic beam in mammalian tissues compared to the expected ultrasonic beam [1], [2], [3], [4], [5]. The causes of beam broadening were referred to as aberrations [6], [7], [8], and were initially characterized as distortion of the wave due to tissue or sound speed heterogeneity. Since the early days of the exploration of aberration, aberration in diagnostic ultrasound is more broadly understood as any deviation in wave propagation from the expected propagation in a homogeneous sound speed medium. This concept of aberration is typically limited to the forward propagation of the wave from source to receiver, meaning it does not include distortions from wave interference from multipath scattering (e.g. reverberation) or other scattering sources. It is also important to recall that diagnostic ultrasound requires two-way propagation of the wave, and therefore both the transmitted and reflected waves experience aberration.

Clinically, phase aberration can reduce the viability of ultrasound for diagnosis and treatment in a multitude of ways. Refraction between subcutaneous fat and muscle is one of the dominant factors behind image degradation due to aberration [9]. Therefore, ultrasound applications involving overweight and obese patients, a significant percentage of the world’s population, stand to benefit from aberration correction [10]. Abdominal, cardiac, breast, and fetal applications are the most common ultrasound imaging applications and all are affected by this poor *body habitus*. In applications such as diagnostic and therapeutic transcranial ultrasound, where the effect of subcutaneous fat is less pronounced, the skull contributes heavily to aberration due to the large sound speed difference in the skull bone [1].

Frequently, an aberrated wavefront consists of phase or time delays that are associated with the localized changes in the sound speed of the medium due to tissue heterogeneity. These errors are frequently referred to in the diagnostic literature as time-delay errors, arrival time errors, phase errors, aberration law and various similar terms. In addition to time-delay errors, amplitude distortions in the wavefront can also be present due to diffraction, refraction, and attenuation resulting from variations in tissue properties across the lateral spatial domain of the wavefront [11].

**Fig. 1:**
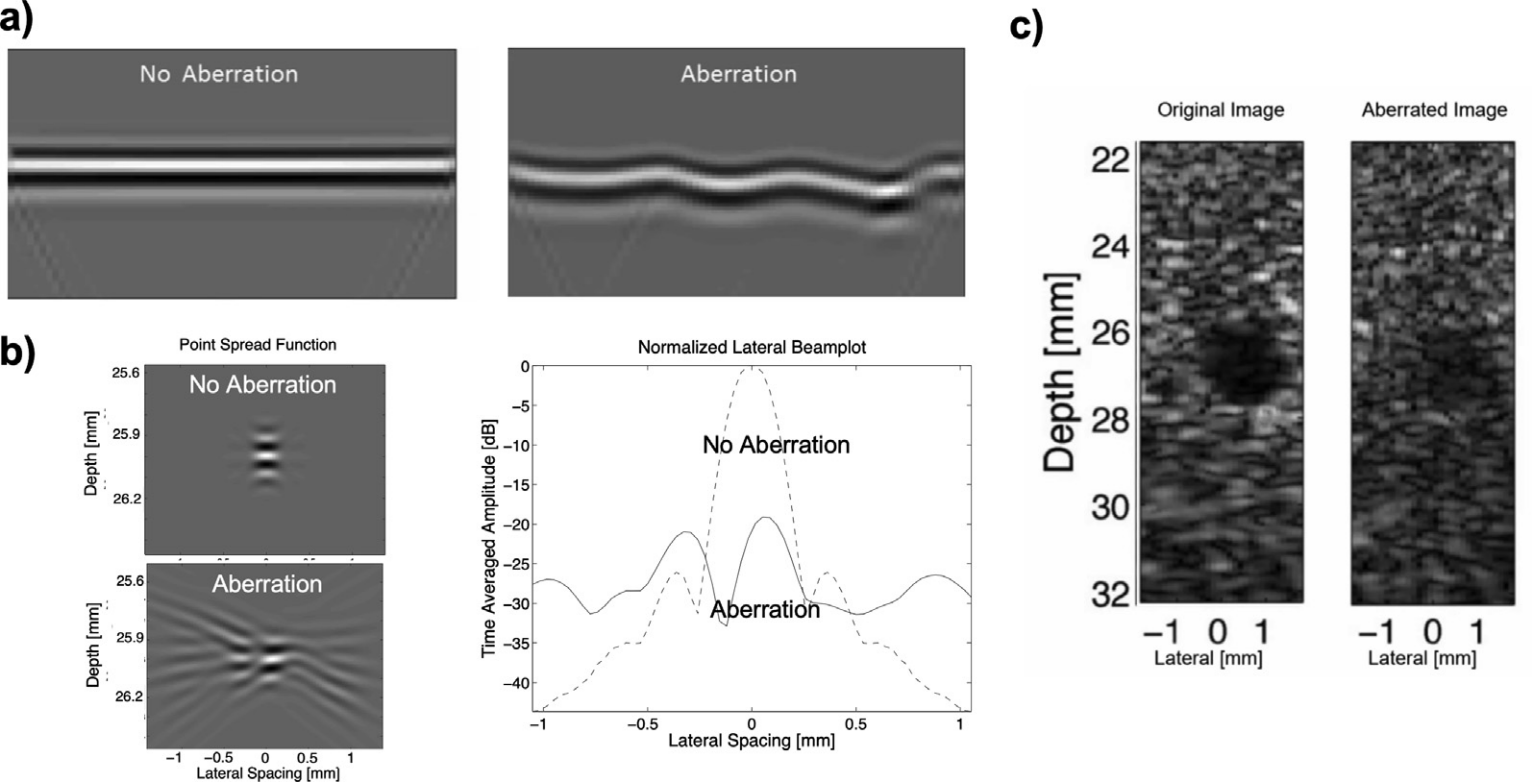
a) Aberration in a geometrically time-delayed ultrasound wavefront, characterized by phase delay and amplitude variation along the transducer aperture. Aberration distorts the point-spread function b) and reduces contrast in the ultrasound image c).

Fig. 1 compares received unaberrated and aberrated pulse-echo waveforms produced by propagation of a focused wave from a transducer to a point reflector and back to the transducer. Time-delays necessary to account for geometrical path-length differences have been applied to the waves, assuming a propagation velocity of 1,540 m/s. In the case of the unaberrated wave, these “focusing delays” result in a “straight” appearance from left to right across the transducer elements as the waves are perfectly aligned. The aberrated echo waveform, however, shows prominent distortions in the form of time-delays, which produce a “wavy” appearance compared to the straight appearance of the unaberrated wave. In addition, the amplitude of the aberrated wave is lower than the amplitude of the unaberrated wave (i.e. the peaks are a brighter white and darker black in the unaberrated wave compared to the aberrated wave). There are also a few minor amplitude variations and artifacts that appear in the aberrated wave that are not present in the unaberrated wave.

The time-delay and amplitude errors of aberration effectively cause a distortion of the point-spread-function. The distortion typically manifests as a broader main lobe with increased side lobes (Fig. 1b), although effects such as a split main lobe and steering errors can also occur. The broader main lobe results in a loss of resolution, while the elevated side lobes increase scattering from side lobes (i.e. off-axis scattering), which decreases target contrast and visibility (Fig. 1c). Effects such as split main lobes and steering errors can result in image artifacts. In addition to the distortion of the point-spread-function, Trahey et al. [12], [13], showed that the brightness of the echo was proportional to the root-mean-square of the point-spread-function. As demonstrated in Figs. 1b, the root-mean-square of the distorted point-spread-function is much smaller than the undistorted point-spread-function, resulting in a darker overall image under aberration compared to the image without aberration (see Fig. 1c).

It was recognized relatively early on that higher imaging frequencies were more greatly affected by aberration than were lower frequencies [14]. This was observed to be a result of the size of the time-delay relative to the wavelength of the echo [15], which results in greater destructive interference at higher frequencies. For example, a fixed time delay of 25 ns would correspond to a phase of roughly π/20 radians at 1 MHz, but would correspond to a phase of roughly π/2 radians at 10 MHz.

It has been of significant interest to the field to correct, or at least mitigate, aberration and its effects. In this review, we explore the field of aberration correction, from the early stages to current and future directions, as it relates to diagnostic pulse-echo ultrasound of soft tissues. Although related, aberration correction in the fields of therapeutic ultrasound and ultrasound computed tomography are not discussed. In the following review of aberration correction, sampling of aberration is reviewed in Section 2 because it pertains to the quality of the correction. In Section 3, models of aberration are reviewed; the models of aberration have a significant influence on the correction techniques that have been developed. In Section 4, early aberration correction techniques are reviewed, and in Section 5, current and future directions of aberration correction techniques are discussed.

## 2 Sampling of aberration

Efforts to measure and correct aberration and its effects on image quality began appearing in the 1980’s [16], [17], [18], [19], [20], [21], [15]. These initial measurement techniques were primarily concerned with measuring the phase or time delays imposed by sound speed errors, and thus referred to these errors as phase aberrations. However, both phase aberration and time-delay errors are used interchangeably throughout the literature and here in this review. More broadly, aberration can be considered a frequency-dependent phenomenon that affects both the speed and amplitude of the propagating ultrasound wave [22], [23]. Therefore, measurement of the phase and amplitude of the distortion more accurately captures the aberration.

**Fig. 2:**
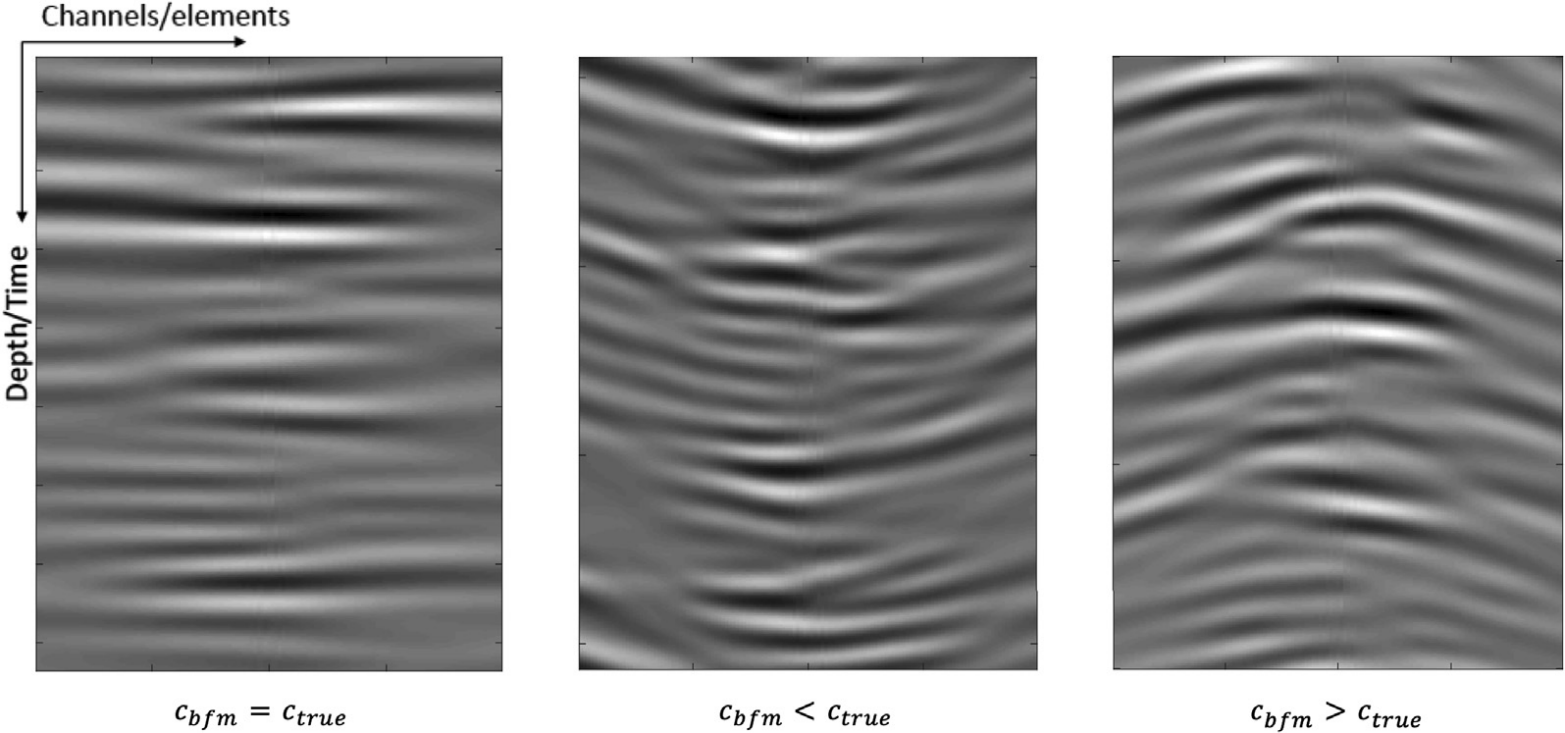
Effect of beamforming sound speed error, $c_{\mathrm{bfm}}$, on channel signal alignment after applying geometric focal delays. All of the signals shown are received channel signals (with synthetic transmit focusing) from a diffuse scattering medium with sound speed $c_{\mathrm{true}}$. (left) The channel signals are aligned correctly when $c_{\mathrm{bfm}}=c_{\mathrm{true}}$, but (middle) display an upward curvature when $c_{\mathrm{bfm}}<c_{\mathrm{true}}$ and (right) a downward curvature when $c_{\mathrm{bfm}}>c_{\mathrm{true}}$.

Overall, the phase aberrations induced by sound speed errors can be classified as a result of global or local sound speed errors. A global sound speed error occurs when the beamformer under- or overestimates the sound speed of the scattering medium, which leads to depth-dependent time-delay errors that appear as a visible curvature in the received signals after geometrical focal delays have been applied (Fig. 2). The errors are depth-dependent because the time-delay errors accumulate as the wave propagates deeper into the medium. Local sound speed errors occur when tissue inhomogeneities, such as pockets of differing tissue types, cause distortions in localized areas of the wavefront, resulting in the “wavy” appearance shown in Fig. 1a.

### 2.1 Components/metrics of aberration

**Fig. 3:**
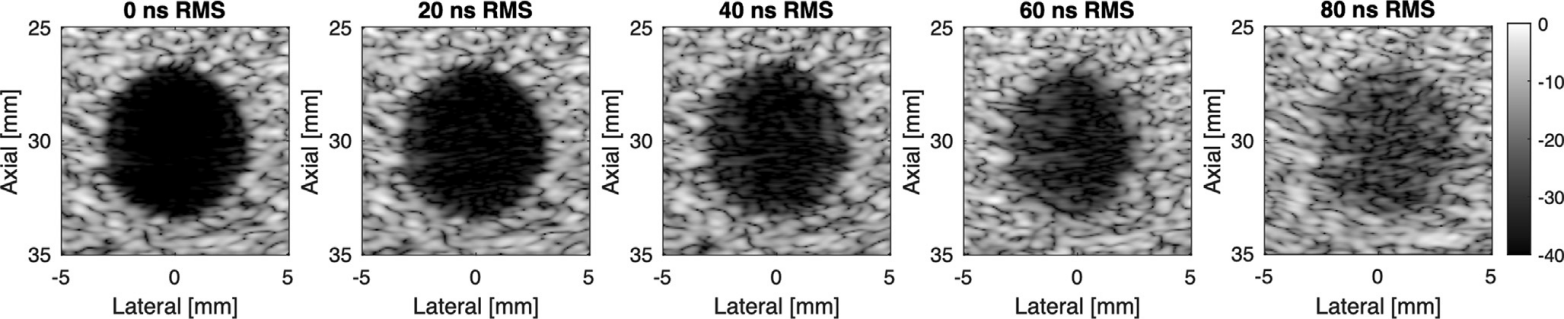
Field II was used to simulate backscatter from this anechoic lesion target with a 96-element linear array transducer (0.215 mm pitch, 3.5 MHz, 70% fractional bandwidth). Aberrators with a full-width at half-maximum correlation length of 2.60 mm were simulated with aberration strengths of 0, 20, 40, 60, and 80 ns RMS error.
**Correlation Length:** The correlation length of the aberrator is computed from the full-width at half-maximum (FWHM) of the autocorrelation of the time-delay errors across the ultrasound transducer array and is typically given in units of millimeters. This parameter describes the spatial frequency content of the phase aberration, or the rapidity of the time-delay oscillations across the ultrasound transducer. As the correlation length of the aberrator decreases, higher spatial frequencies are introduced into the aberrator, resulting in greater distortion in the image.
**Steering Error:** This parameter describes how far the phase aberration steers the focus of the beam away from the intended direction. This can either be measured as the number of degrees shifted away from the main beam axis or number of millimeters shifted away from the intended focal location. Although a steering error does not necessarily imply a distortion of the beam, larger steering errors can induce more significant image artifacts.
**Energy Level Fluctuations:** Although amplitude aberrations are also present, they were infrequently measured or reported in the literature. However, Hinkelman et al. [24] introduced the concept of energy level fluctuations to describe the severity of the amplitude variations. Energy level fluctuations are computed as the RMS value of the square of the amplitude of the wavefront across the ultrasound transducer and is typically given in decibels [25]. A larger energy level fluctuation corresponds to greater image distortion.

Because the effect of phase aberration (and aberrations in general) can vary depending on the distribution of the aberration over the ultrasound transducer, aberrations are typically described by the following parameters:

**Strength:** The aberration strength is typically computed as the root-mean-square (RMS) of the time-delay errors, and provides an indication of the severity that the phase aberrations have on the image quality. A larger RMS error indicates stronger aberration effects and worse image quality. In diagnostic ultrasound, it is generally computed in units of nanoseconds RMS, although some papers utilize units of radians or degrees when the error is computed as a phase. Fig. 3 shows the degradation in the image quality of an anechoic lesion target as the aberration strength of the aberrator is gradually increased from 0 to 80 ns RMS.

Initial in vivo exams (n = 6) examining the magnitude of phase aberration measured aberration strengths of around 20 to 50 degrees RMS in the abdomen, corresponding to 17 to 32 ns RMS, where the latter was associated with poor image quality of the liver [19]. Subsequent studies in autopsy specimens examining phase aberration across the abdominal wall measured phase aberration strengths in the range of 25.6 to 75.9 ns RMS, correlation lengths in the range of 2.60 to 11.11 mm, and energy level fluctuations in the range of 2.74 to 3.70 dB RMS [24], [26]. In simulations of 6 abdominal wall models, the mean energy level fluctuation were 3.50 dB RMS [26]. The abdominal wall simulations also revealed that, from a total 26.7±4.3 ns RMS aberration, 17.6±2.1 ns RMS was introduced by subcutaneous fat and 9.1±3.2 ns RMS was introduced by muscle layers. Spatial correlations suggested that the largest arrival time fluctuations are induced by propagation through large-scale inhomogeneity such as fatty regions within muscle, while amplitude variations result form scattering of smaller inhomogeneity such as septa within subcutaneous fat [27]. In a chest wall autopsy study [28], phase aberration strengths of 21.3 ns RMS were produced by 16 different samples of intercostal tissues.

The average phase aberration strengths in several in vivo studies in human breast tissue were measured to be 36.1 [29], 55.3 [30], 22.9 [31] and 28.0–35.8 ns RMS [32]. In breast autopsy specimens, phase aberration strengths of 66.8 ns RMS were measured [33]. The correlation lengths in these measurements ranged from 2.1 to 5.2 mm [29], [30], [33], [32], had energy level fluctuations of 5.03 dB RMS on average [33], and had steering errors from 1.1 to 1.5 degrees [29], [30] or beam displacements of up to 10 mm [34], [35]. It is worth noting that the studies measuring aberration by a receiving transducer on opposite sides of the tissue from a transmitting transducer [35], [29], [30], [34], [33] showed significantly greater aberration strengths and effects compared to the measurements from pulse-echo ultrasound [36], [31], [32]. Patient cohort statistics of these aberration studies are summarized in Table 1.

**Table 1:** In vivo sampling of aberration in human breast tissue.

Reference	n	Age	Measurement	Frequency	Aberration
		(years)	Type	(MHz)	(ns RMS)
[29]	22	45 (average)	pitch-catch	4.2	36±15
[30]	7	40-59	pitch-catch	4.3	55±14
[31]	8	n.a.	pulse-echo	8.5	23±8
[32]	3	55-70	pulse-echo	6.5	28±6
	4	24-40	pulse-echo	6.5	36±12
[36]	12	n.a.	pulse-echo	n.a.	25±14
	12	n.a.	pitch-catch	n.a.	60±23
n.a.: not available					

### 2.2 Second harmonics in aberration estimation

Tissue harmonic imaging, or imaging at the second harmonic frequency, was rapidly adopted onto ultrasound scanners in the late 1990’s due to its superior image quality compared to conventional imaging using the fundamental frequency. Although initially proposed as a possible imaging method that was less affected by aberration [37], it was quickly shown that aberrations observed at the second harmonic frequency were identical to those observed at the fundamental frequency [38], [39], [40] and that the second harmonic amplitude was weakened by the aberration [41], [42]. This implied that tissue harmonic imaging was more susceptible to degrading effects of aberration and that aberrations were better sampled at the fundamental frequency. However, because second harmonics have an effective transmit apodization that is different than the fundamental frequency, the correlation of second harmonic echo signals could be optimized to obtain more accurate measurements of phase aberration by altering the transmit apodization of the fundamental frequency [43], [44], [45].

### 2.3 Transducer technology for aberration measurements

Whatever the cause or origin of an aberrated ultrasound wavefront, its phase aberration profile is effectively measured by the transducer array. After delays are applied (to account for geometrical path-length differences and based on a reasonable average sound speed estimate), the aberration profile from local sound speed errors can be considered a near-zero-mean signal across the face of the transducer array. The center-to-center spacing (‘pitch’) of the array elements is effectively a lateral spatial sampling period of this aberration profile signal. Therefore, by the Nyquist-Shannon criterion, the element pitch sets an upper limit on aberrator variability (correlation length) that can be adequately sampled and characterized by a given array.

Phase aberration can occur anywhere and along any direction over the area of an assumed-spherical echo wavefront, but most ultrasound imaging arrays sample this effect in only one lateral dimension. Most ultrasound probes are “1D” linear, curvilinear, or phased arrays that are often diced on a 0.5λ-1λ pitch in azimuth, with a single, comparatively large (tens of wavelengths) element in elevation. With such an array, an aberration with a correlation length as small as 2λ may be theoretically sampled adequately in azimuth, but in reality, the measured aberration is averaged or “smeared out” by each element’s large single sample in elevation [46], [47] and affects accurate measurement and correction of the aberrator [48], [49]. Lacefield and Waag [49] recommended that the pitch of the array (either in elevation or azimuth) be no more that 50% of the correlation length of the aberrator in order to adequately sample the aberration.

The need for adequate aberration sampling in the elevation dimension can be addressed using arrays that are also segmented and electronically controllable in elevation, which are known as 1.75D or 2D “matrix” arrays, as described by Thomenius [50]. To date, several researchers have investigated the impact of 1.75D or 2D arrays for phase aberration estimation and correction on both a theoretical and practical basis. In the late 1990’s, researchers at GE developed a 3.3 MHz, 1.75D array probe with 6×96 (576 total) independent elements on a 1.5 mm × 0.6 mm pitch, respectively [51]; the probe was connected to a 128-channel beamformer, flexible multiplexer, and an auxiliary multiprocessor computer. This probe and system were used to implement an iterative beamsum-channel correlation method, to demonstrate adaptive correction of 2D time-shift aberrations during real-time scanning of both phantoms and in vivo targets [52], [53].

In the early 2000’s, researchers at the University of Rochester created a 3.0 MHz, 2D array with 80×80 (6400 total) independent elements on a 0.6 mm × 0.6 mm pitch, with all elements addressable for transmit and receive via synthetic aperture techniques [54], [55]. This array was used to investigate design tradeoffs in a multi-row array’s ability to estimate arrival time fluctuations through distributed aberrations and analyze 2D spatial coherence functions as a measure of aberration [49], [56].

From the early 2000’s through mid-2010’s, researchers at Duke University implemented phase aberration correction methods on a variety of 1.75D and 2D probes. The first was a 8.5 MHz, 1.75D array probe with 3×80 (240 total) independent elements; this array was used to analyze time shift estimates in conventional and harmonic echo data from the breast [31]. The second was a 6.5 MHz, 1.75D array probe with 8×128 (1024 total) independent elements on a 1.5×0.2 mm pitch; this array interfaced with a system that allowed different individual rows to be active on transmit and receive, enabling synthetic aperture beamforming in elevation [32], [57]. The third was a 9 MHz, 1.75D array probe with 8×96 (768 total) independent elements on a 1.0×0.2 mm pitch; this array was used to analyze and suppress off-axis scatterers’ contribution to aberration, and to investigate imaging tradeoffs with adaptive phase correction and spatial compounding [58], [59], [60], [61], [62]. The fourth was a 2.5 or 3.5 MHz, partially-sparse 2D array with 440 transmit elements including 256 receive elements, within a 40×40 grid with a 0.35×0.35 mm pitch; this array was used to implement phase-aberration correction for transcranial 3D B-mode, Doppler, and contrast-enhanced imaging [63], [64]. The fifth was two 2.5-MHz, partially-sparse 2D arrays, each with 512 transmit elements including 256 receive elements, within a 32×32 grid with a 0.35×0.35 mm pitch; these arrays were used in a pitch-catch configuration to perform phase-aberration correction on multiple isoplanatic patches (see Section 3.3) for transcranial 3D B-mode and Doppler imaging [65], [66].

While each of the works mentioned above show the potential to significantly improve image quality through phase aberration correction on 1.75D and 2D arrays, it should be noted that each also encountered system limitations, in terms of data acquisition channels and processing speed. New approaches to ultrasound acquisition and processing and new system architectures may enhance the practical use of multi-dimensional transducer arrays for phase aberration correction in the future.

## 3 Aberration models

### 3.1 Near-field phase screen model

Models were developed and refined over time to represent aberration and form a basis for methods to correct for the modeled causes of aberration. An early model for aberration was the near field phase screen model [19]. This relatively simple model assumed near field velocity inhomogeneities to be the primary source of aberration in imaging systems due to the cumulative nature of aberration errors. Therefore, aberration was represented as a thin, irregular screen at the surface of the transducer. A single set of aberration delays for each element could then be used for correcting the aberration across the image [20], effectively meaning that the model had an infinite isoplanatic patch (see Section 3.3). Although this model accounts for a significant source of aberration close to the transducer [19] and was widely adopted for aberration correction methods, the near-field phase screen model did not account for amplitude variations and propagation effects through inhomogeneous media, thereby yielding inconsistent modeling of aberration.

### 3.2 Distributed models

To accommodate propagation effects and amplitude errors that were lacking in the near field phase screen model, distributed aberration models were developed. The following models describe numerous strategies to model the more complex phenomena induced by inhomogeneous media.

#### 3.2.1 Mid-field phase screen models

**Fig. 4:**
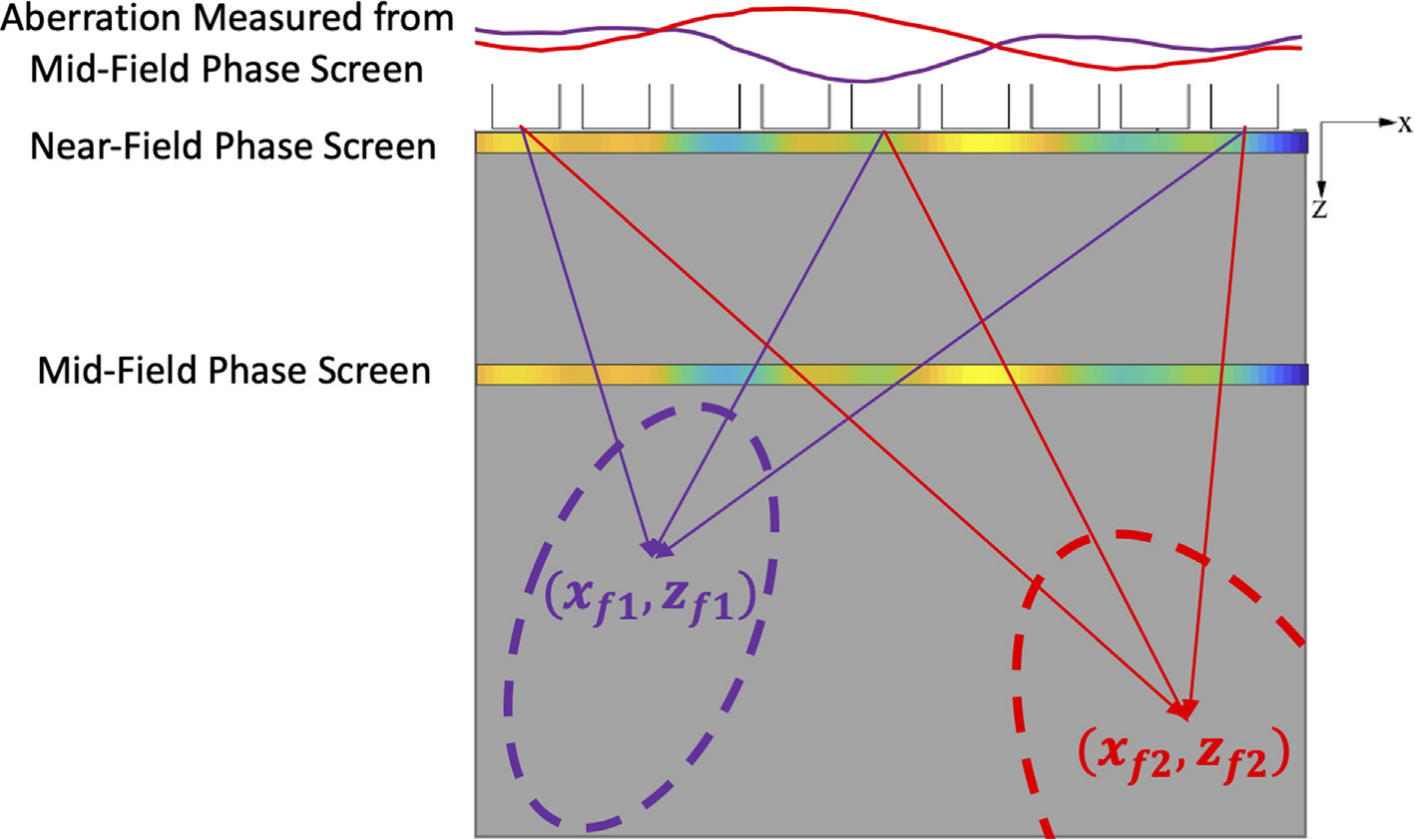
Mid-field vs. near-field phase screen. Ray paths between 3 transducer elements and 2 imaging points $\left(x_{f1},z_{f1}\right)$ and $\left(x_{f2},z_{f2}\right)$ are shown here. Note that ray paths to each imaging point intersects the mid-field phase screen at different locations, inducing a different set of aberration delays at each imaging point. The rough region over which each set of aberration delays would be applicable (also known as the *isoplanatic patch*) are indicated by the dotted circles shown. This is not the case with the near-field phase screen model, which would have an isoplanatic patch with a single set of aberration delays that covers the entire imaging region.

Unlike the near-field phase screen model, the mid-field phase screen model assumes that this thin film occurs at some depth away from the transducer surface (see Fig. 4) [25], [16], [17]. Because the phase screen is no longer at the transducer surface, the wavefront must propagate from the location of the phase screen to the transducer after undergoing phase (or time) shifts. As portions of the wave advance or slow relative to the other parts of the wave, a quasi-aperture is developed, which induces localized diffractive (self-interference) effects in the propagating wave [67], [68], thereby creating amplitude errors and other effects. Unlike the near-field phase screen, there is no set of time delays that can ideally account for the aberration in order to reconstruct an ideal main beam, and some form of compensation to amplitude is required. In addition, the aberration can vary with the position of the focal point because the path of propagation from each transducer element to the focal point will intersect with the phase screen at different locations or times.

More sophisticated models of mid-field phase screens incorporated a propagation step (based on the angular spectrum method) to and from the aberrating phase screen to account for these diffractive aberration effects [69]. The mid-field phase screen was also shown to be an effective model for aberrations throughout the image in cases where the sound speed variation responsible for the aberration was not contained within a single thin film, but a set of multiple mid-field phase screens [70], [71].

#### 3.2.2 Filter-based models

Distributed aberrators can often distort the shape of the transmitted waveform in addition to simply delaying or advancing the ultrasound signal. These additional distortions are the result of diffraction when the transmitted waveform interacts with the aberrator. These diffractive effects can either be modeled as a spatially-varying phase and amplitude response at each frequency [70], [72], [73], [74] or alternately as a spatially-varying time-domain convolution with a filter [75]. These filter-based models generally use spatially-varying impulse responses that can also vary for each transducer element. This modeling approach requires special care to ensure that the estimated impulse response varies smoothly over isoplanatic patches.

#### 3.2.3 Direct computation of aberration via sound speed models

Although the previously discussed models of aberration have been used to simplify the complex nature of aberration, more direct models of aberration can be generated by simulated wave propagation or time-delay computation through numerical models of heterogeneous tissue incorporating the sound speed of the tissues. These models can provide more accurate observation and exploration of the aberration present in a medium; however, application of these models for in vivo aberration correction requires the estimation sound speed from in vivo tissue, which is not a trivial task.

Wave propagation models can account for diffraction and refraction-related effects on the ultrasound signal as a result of spatially-varying sound speed in the medium [11], [22], [76], [77], [78]. Wave propagation models have included finite difference time domain implementation of the linear wave equation [11], [22] and the nonlinear wave equation [78], pseudospectral implementation of the nonlinear wave equation [79], and variations of the angular spectrum method [76], [80]. Because wave propagation is parameterized by the sound speed, these models require a model of the sound speed from the tissue. Although this has easily been implemented using numerical models derived from histology samples [11], [22] or other medical imaging modalities [79], application to aberration correction requires estimation of sound speed from the tissue.

**Fig. 5:**
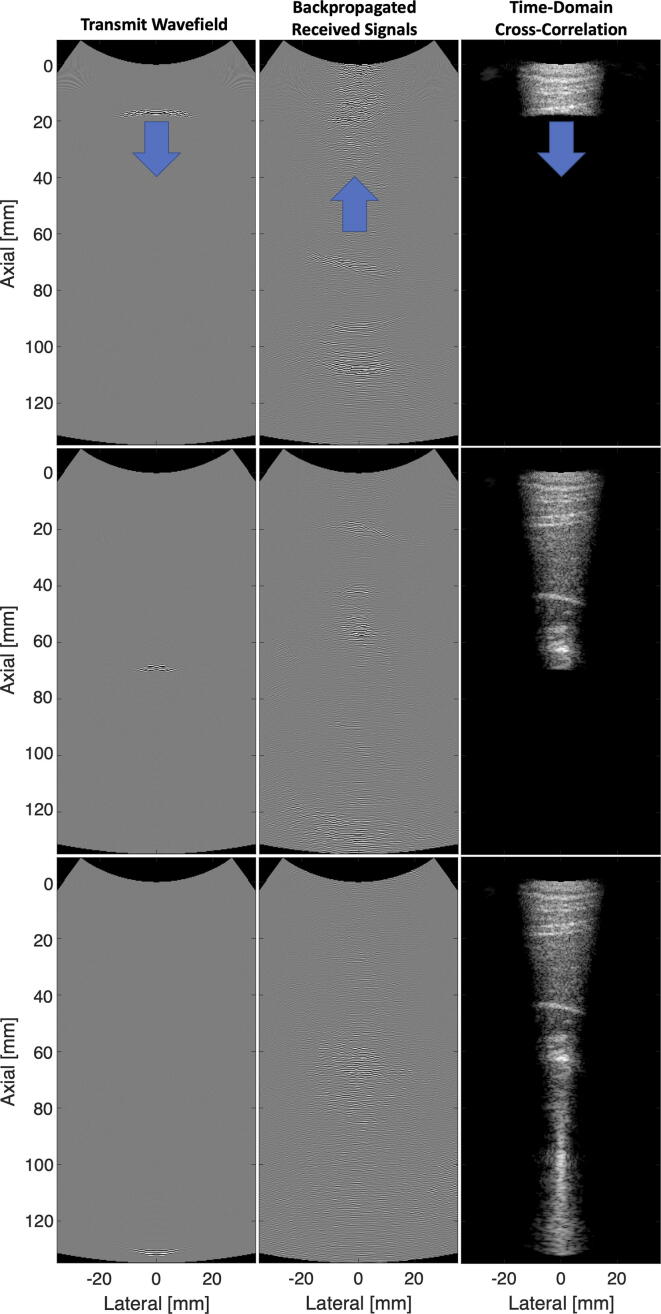
Image reconstruction by time-domain correlation of a focused transmit wavefield and a backpropagated receive wavefield. Channel data was acquired using a Siemens 5C1 on the abdomen of a healthy human volunteer [80].

Additional wave propagation methods include the Fourier split-step methods [81], [82], [83], [84]. These methods can be interpreted as a series of mid-field phase screens with depth parameterized by the sound speed in the medium (rather than a full 2D parameterization of sound speed), which is essentially a depth-wise extension of the mid-field phase screen model to fully account for the spatially-varying profile of the sound speed in the medium. Fig. 5 shows an example of the Fourier split-step wave propagation approach used to reconstruct an ultrasound image based on a sound speed distribution of the medium [80], [85].

**Fig. 6:**
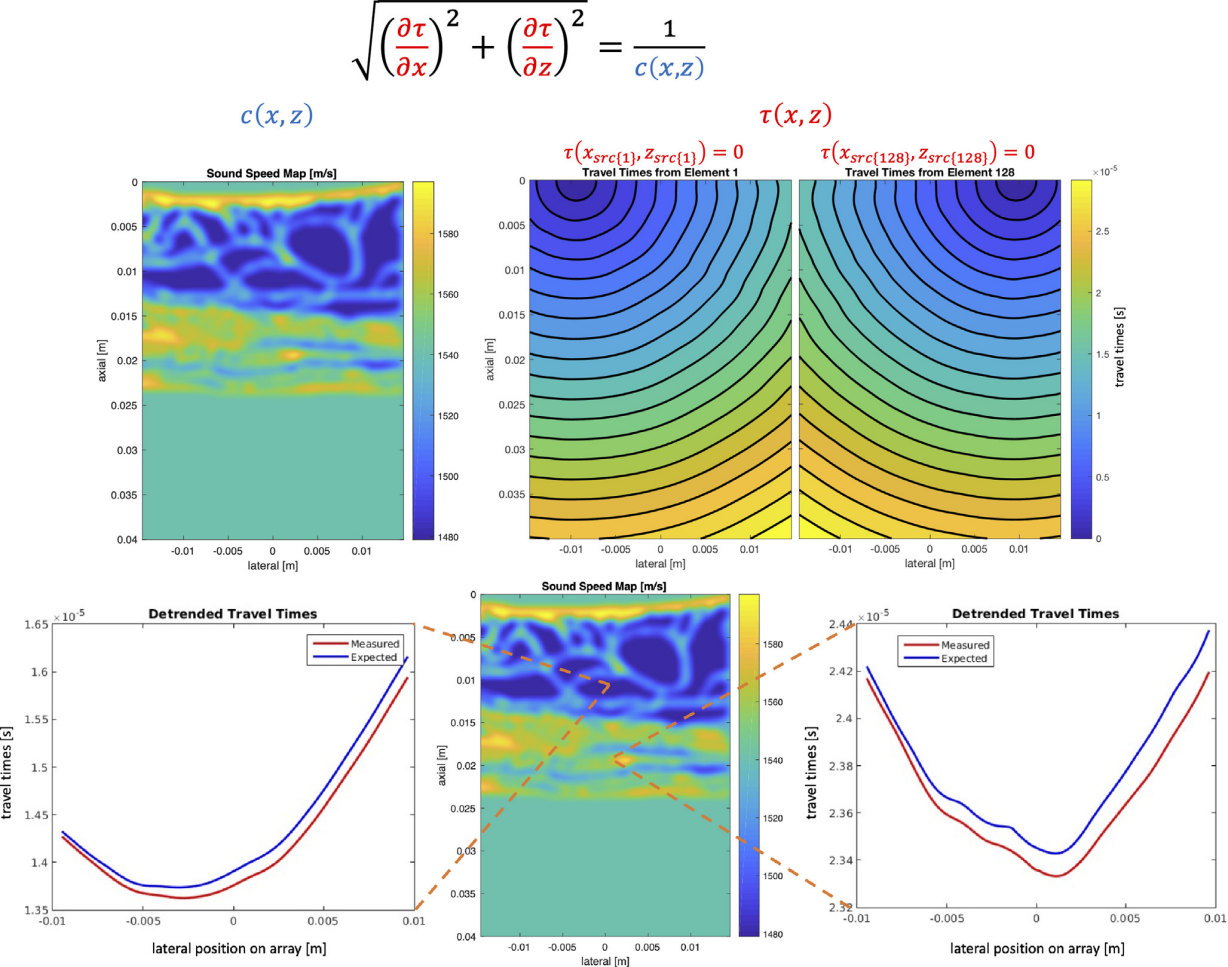
Modeling time-of-flight τ(x,z) in a spatially-varying sound speed medium c(x,z) using the eikonal equation (top). Time-of-flight between two imaging points and the array elements as predicted by the eikonal equation and estimated based on cross-correlation of signals across the array (bottom). The time-to-depth ambiguity is responsible for the offset between the estimated and predicted aberration delays.

As an alternative to simulated wave propagation, which can have a heavy computation burden, time-of-flight models can be used to capture the arrival times of the wavefront based on numerical sound speed models of tissue. The time-of-flight distribution for each transducer element to the scattering locations can be modeled using straight ray tracing or the eikonal equation. Straight ray tracing simplifies the computation by computing the time-of-flight along the direct straight line to the scattering location, ignoring refraction. The assumption here is that, because the variation in sound speed among most soft tissue is relatively small (less than 10%), the effects of refraction are negligible. Alternatively, the eikonal equation is a nonlinear partial differential equation that models refraction and is an approximation to the heterogeneous wave equation. Fig. 6 shows an example of the eikonal equation applied to a numerical sound speed model of abodominal tissue to compute the arrival times of the wavefront at various scattering points in the medium. The eikonal equation shows that the arrival times vary depending on the scattering location in the medium, depicting it’s ability to model distributed aberration. The arrival times given by the eikonal equation are compared to arrival times estimated by cross-correlation, demonstrating the ability to compare the performance of aberration estimation/correction techniques to the ideal arrival times.

### 3.3 Isoplanatic patch

In the approximation of the near-field phase screen aberrator, a single set of aberration delays is responsible for the distortion in the whole medium. Thus, a unique set of time delays can be implemented in the beamformer to compensate for the aberration delays. However, in biological media, the aberrating layer thickness is non-negligible and the infinitesimally thin screen is frequently insufficient to describe the aberration at all points in the medium. The physical imaging space over which the effects of the aberration remain consistent, or alternatively, the physical region over which the wavefront distortion remains constant, is referred to as the isoplanatic patch [15]. The isoplanatic patch size characterizes the spatial stability of the aberrator and decreases when the aberrator complexity increases. For example, an aberrator that is well-characterized by a thin phase screen at the transducer surface has a large, and theoretically infinite, isoplanatic patch size, while a mid-field thin phase screen will have a much smaller isoplanatic patch size. The isoplanatic patch also depends on the sampling capabilities of the system as well as the aberration estimation approach.

In general, the isoplanatic patch is systematically larger in the axial direction than in the azimuthal direction. Measurements for abdominal tissue [86], breast, liver, thyroid [87], or skull bone [88] have illustrated the diversity and specificity of isoplanatic patch sizes - from a few wavelengths to several centimeters. Determining the isoplanatic patch size is a major preamble to any phase aberration correction approach based on delay compensation. Patch size will define the spatial sampling of the aberration correction and thus, its computational efficiency (see isoplanatic patches in Fig. 4).

## 4 Early aberration correction techniques

### 4.1 Challenges in aberration correction

As will be observed through this and the following sections, there are several ongoing challenges with aberration correction that are consistent across all aberration correction techniques. These challenges include (a) reconstructing coherent phase information from diffuse scattering signals, (b) achieving accurate time estimates from unknown reflector locations, (c) identifying signals with enough signal-to-noise-ratio at larger tissue depths, (d) filtering out low-quality phase or time-delay estimates, and (e) handling anechoic regions that contain no usable signals for estimation. Although each aberration correction technique may focus on one or two of these problems, solving all of these challenges is equally important important for real-time and clinically usable aberration correction because each has the potential to render aberration correction useless if not appropriately addressed.

### 4.2 Near-field phase screen correction

Some of the earliest aberration correction techniques relied on the near field phase screen model of aberration. In this case, to compensate the beamformer and restore image quality, an estimate of the near field phase screen was required. Because of the infinite isoplanatic patch size, only a single set of time delays from the aberrator was required. Flax and O’Donnell [20] proposed a nearest neighbor cross-correlation technique in which signals from neighboring receive elements were cross-correlated as a function of time-lag. The time-lag (or time-delay) that maximized the cross-correlation function yielded the aberration delay for one of the elements. Because the number of cross-correlations was one less than the total number of array elements, one of the elements was named the reference element, which was given a time-delay value of zero. Thus, all other time-delays computed in this method were relative to the time-delay of the reference element. It is interesting to note that cross-correlation between signals from adjacent elements was proposed just a few years earlier to determine relative time delays for point target sources only [89]. The key observation in Flax and O’Donnell [20] was that the signals reflected from diffuse scattering media are highly correlated on neighboring transducer elements, a property described later under the adaptation of the van Cittert-Zernike theorem to ultrasound wave propagation [90].

To fully compensate the beamformer, these estimated time-delays need to be applied to both the transmitter and receive delays. In addition, because compensation of the beamformer’s transmit time-delays improves the underlying point-spread-function of the imaging system, this (and other near field correction) techniques could be performed iteratively to yield further improvement. In each iteration, the estimated aberration is applied to the transmit time-delays and a new pulse is transmitted. The resulting aberration estimate from this new transmission is more accurate than the previous estimate. Iterative correction is not necessarily limited to near-field correciton techniques; this same process can and has been applied with other aberration correction technique, including eigendecomposition and distributed aberration techniques [91], [92], [93], [53], [73], [62].

In addition to cross-correlation of neighboring elements, there are a wide variety of proposed near-field correction methods. The speckle brightness technique [15], [94] relies on the maximization of the mean speckle magnitude in a target ROI to determine the correct time-delay for any given element. Subsequent iterations of this technique include the speckle-lookback technique, which utilized subsets of corrected time-delays in the calculation of the speckle brightness when determining the time-delay of a specific element [95] and aberration correction from moving speckle targets, which utilize speckle from blood [96], [97].

The beamsum method is another method of utilizing cross-correlations to estimate aberration delays, although the individual channel signals were correlated to the total beamformed signal [98]. The beamsum method was proposed in order to eliminate the need for a reference element, since the beamsum acted as the reference value. An improvement to this method was introduced by Krishnan et al. [92], where the beamsum method was combined with nearest-neighbor cross-correlation to reduce the underestimation bias and cumulative delay error that resulted from nearest neighbor cross-correlation and the variance that resulted from beamsum due to low correlation values. The delay estimates were retained from beamsum if the correlation exceeded a threshold based on the statistics of the beamsum correlation coefficients, and the remaining delays were filled in using the nearest-neighbor correlation method.

Techniques using eigendecomposition of cross-correlation matrices have also been proposed for aberration correction. Varslot et al. [99] and Måsøy et al. [73] perform aberration correction by maximizing the expected energy after beamforming, which occurs when transmitting the eigenfunction corresponding to the largest eigenvalue for a spatial correlation function. Aberration correction performed this way is equivalent to using a phase screen near the transducer that adjusts for both the time delay and amplitude based on the amplitude and phase of eigenvector or eigenfunction components. Måsøy et al. [73] showed mathematically that this type of correction is equivalent to the beamsum method. Interestingly, earlier iterative time-reversal methods [100], [101] (Section 4.3) can also be described using eigenvector or eigenfunction formulations, where transmitting different eigenvectors or eigenfunctions result in distributed aberration correction for scatterers of different intensities or homogeneities.

**Fig. 7:**
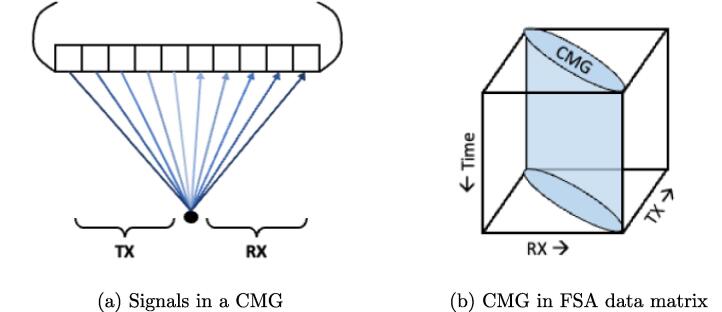
Illustration of common midpoint gathers (CMG). The signals from the transmit and receive pairs in (a) constitute a common midpoint gather, and when represented in a full synthetic aperture (FSA) data matrix (b), the common midpoint gather signals lie along the anti-diagonal of the matrix.

Another approach to determining near-field aberration delays utilize signal redundancy through common midpoint gathers. Common midpoint gathers are received signals from transmit-receive element pairs that share the same midpoint, as shown in Fig. 7, and the correlation between any two of these signals is very close to 1, regardless of target distribution [102], [103]. However, with phase aberrations, these common midpoint gathers shift. In Rachlin [104], a radiointerferometry redundancy method was adapted to ultrasound by cross-correlating common midpoint gathers to find the relative delays that maximized their correlation. A linear equation relating the relative delays to the final applied phase aberration delays was then solved as an overdetermined least-squares problem. However, this method assumed all targets to be in the far-field, while in the near-field, the common midpoint gathers exhibited less correlation. Correction of this method for near-field effects was introduced by Li [102], [105], where a dynamic near-field correction was implemented to align envelope peaks of echoes from a particular direction in order to increase correlation. The corrected signals could then be used to calculate the aberration delays similarly to Rachlin [104].

Similar to Li [102], [105], the translating transmit apertures algorithm is a generalized version of the common midpoint gather that applies to all transmitting and receiving aperture types [45]. In the translating transmit apertures algorithm, the signal generated from any transmit and receive aperture pair (i.e. not just a single element) is theoretically identical to the signal generated from a second transmit and receive aperture pair where the second transmitter and receiver are translated an equal distance in opposite directions relative to the first transmitter and receiver. The primary advantage of this algorithm is to increase the correlation between element signals to improve estimation and correction of aberration delays.

It is worth noting that many of the techniques and algorithms above are able to be combined. For example, the cross-correlation approach in Flax and O’Donnell [20] was combined with Rachlin’s least squares approach [104] and the property of phase closure introduced by Freiburger et al. [30] to enable a multi-lag least squares algorithm [25] to improve the estimation of aberrating time delays.

Early methods of real-time correction relied on the aforementioned near-field phase correction techniques to rapidly correct aberration for clinical use, primarily because ultrasound systems were limited in their ability to incorporate more sophisticated aberration correction techniques. Specifically, the ultrasound systems could only incorporate compensation to the time-delays, often in a limited fashion such as compensation with a single set of delay values.

Initially, real-time methods were implemented for 1D transducer arrays [94], [97]. Delays used for aberration correction in these systems maximized target ROI brightness [94] and moving target brightness [97]. In order to improve aberration estimates, real-time correction expanded to using 1.75D arrays [52], [53], [58], [62] and later 2D arrays [63], [64], [106]. Many of these methods [58], [63], [64], [106] implement multi-lag, least-squares cross-correlation [31], [107] to estimate aberration delays for multi-dimensional arrays, while a beamsum method was used in [52], [53]. These early real-time correction algorithms took approximately 100 to 300 ms to compute delay profiles.

### 4.3 Mid-field phase screen correction

Given knowledge of the depth of the mid-field phase screen, phase aberration correction can be achieved by backpropagation of the received pressure field via the angular spectrum method [25]. Because the angular spectrum method is applied depth-wise, the mid-field phase screen can be directly applied to the receive pressure field at the depth of the screen before propagating the received pressure deeper into the medium [70].

The main challenge of applying the mid-field phase screen model is estimating the depth of the screen. For point targets at roughly the same imaging depth, there is a closed-form relationship that allows for the estimation of the phase screen depth [71], [75]; however, for a diffuse scattering target, estimation of the depth of the phase screen requires the generation of a virtual source which introduces much greater variability in the estimate. Other methods involve the correlation of the backpropagated received pressure fields to facilitate the measurement of the phase screen depth [68], [76].

### 4.4 Early distributed aberration correction

Some early attempts at aberration correction eschewed models of aberration in favor of adaptive techniques that could accommodate any distribution of aberration. One of the most well-known distributed aberration techniques is the time-reversal technique [108]. In this technique, a focused wave is transmitted into the medium, and the reflected wave is captured by the receivers. A “time-reversal mirror” is then applied, where the captured waveform is reversed in time and transmitted back into the medium. Theoretically, this induces “perfect” focusing capabilities by effectively backpropagating the wave along its diffracted and refracted paths. Despite its ideal focusing capabilities, there are numerous practical challenges to this technique. These include (1) the need for a coherent reflector (e.g. a point reflector) at the focal point in order to differentiate the entire reflected and propagated waveform from scattering from the surrounding soft tissue, (2) the need for an arbitrary waveform generator for each transducer element (an expensive addition to an ultrasound system), and (3) the dependency of the method on a single focal location, thereby requiring re-transmission for every image location.

Later versions of time-reversal proposed some solutions to these issues, such as time-reversal applied to speckle targets [109], which offered an iterative approach that did not require a coherent point reflector. Using the application of the van Cittert Zernike theorem to ultrasound [90], this method consisted of re-focusing multiple speckle targets within the isoplanatic patch in a medium, extracting the focused wavefront, summing the wavefronts from all speckle targets, and retrieving the delay error from the resulting spherical wavefront. The delay obtained was used as an input to re-focus the ultrasound beam (without re-transmitting a pulse because synthetic aperture data was utilized), and the process was iterated to further improve focusing. It is worth noting that this approach did not incorporate the received waveform in the time-reversal as was done with the original formulation by Fink [108], however time-reversal without the full recorded waveform has been shown to be sufficient for aberration correction [110]. Multiple iterations - generally more than 10 - lead to an unaberrated wavefront and provided a fully corrected image. Unfortunately, the need to build an artificial point-like scatterer from several speckle targets comes with an inevitably high computational cost and thus lacked real-time implementation.

Another early distributed aberration correction technique was the parallel adaptive receive compensation algorithm, also known as PARCA [111] and PARCA2 [93]. In the PARCA algorithm, parallel receive beamforming was applied to all imaging locations after a single focused transmit. Parallel receive beamforming in this manner effectively creates an estimated profile of the transmitted beam. From here, an optimization problem is formulated to solve for the set of element time delays that maximize the energy at the mainlobe of the transmitted beam while simultaneously minimizing the energy at the sidelobes of the transmitted beam. A particular advantage of this method over other distributed and mid-field phase screen methods of its era were that it did not require any coherent point reflector to perform aberration correction. However, like many distributed aberration correction algorithms, the method was computationally expensive and lacked real-time implementation.

### 4.5 Corrective power

The evaluation of the corrective ability of aberration correction techniques typically employ a varied set of quality metrics. The literature on aberration correction, both new and old, uses a varied and inconsistent set of metrics, although most metrics relate to some analysis of contrast or resolution. Many of these metrics evaluate the image quality at a localized region in the image (e.g. a cyst or a point reflector). Most recently, however, Rindal et al. [112] introduced the global image coherence, a metric that allows for evaluation of the overall focusing quality over the entire image.

Phase aberration models based on early phase-screen approaches tended to have similar, but limited, ability to correct for phase aberration. In Dahl et al. [62], cross-correlation, speckle brightness, and beamsum algorithms were compared. In post-processed images, the mean contrast-to-noise ratio (CNR) of anechoic lesions in a speckle generating phantom with a silicone aberrating layer was 1.42±0.63, 1.44±0.52, and 1.43±0.54 for each method, respectively, compared to the aberrated image with a mean CNR of 1.22±0.52. In a mid-field phase screen technique using backpropagation and time-shift compensation, Liu et al. [86] achieved a contrast improvement of 2.2 dB using time-shift compensation only (normalized cross correlation), and a constrast improvement of 3.5 dB using backpropagation with time-shift compensation. Although these contrast-based quality metrics are different, they both indicate modest improvement in image quality for near-field and mid-field correction techniques. For iterative correction techniques, Måsøy et al. [73] showed that these techniques converged in image quality in less than 6 iterations, based on the focusing criterion [113] (a localized version of the global image coherence).

In PARCA, Krishnan et al. [93] demonstrate a CNR improvement of an anechoic cyst from 1.73 dB to 3.74 dB. Note that this version of CNR is not the same as calculated in Dahl et al., but like the near-field correction techniques, indicate a modest improvement in image quality. Using the time-reversal technique, Tanter et al. [110], showed that near perfect reconstruction of the mainlobe of the transmitted beam could be obtained down to approximately 20–25 dB below the peak of the mainlobe. However, this technique could only reconstruct a single image point and was not used for imaging.

## 5 Current and new directions

Distributed aberration models overcome the limitations of the near-field and mid-field aberration models. While estimating the parameters of distributed models is complex, continuing improvements in computational power and algorithm development have made distributed aberration correction of full images more tractable.

### 5.1 Distributed aberration correction by spatially-varying near-field phase screen corrections

The benefits of aberration correction on ultrasound image formation are enhanced by the ability to take local variations of the aberration into consideration. Locally adaptive approaches have been recently developed to provide a new level of correction, with the common rationale of estimating the correlation of the images to optimize the focusing.

Chau et al. [114] explored, for instance, a hybrid approach (referred to as LAPAC, for locally-adaptive phase aberration correction) combining multi-lag cross-correlation [31] and the scaled covariance matrix [115]. The scaled covariance matrix is the complex correlation matrix between all channel pairs, where the phase of the correlation matrix is utilized to obtain the time delay estimates. The diagonals of the scaled covariance matrix are used in the multi-lag cross-correlation approach. An overdetermined system of equations can be formed to retrieve the local time delays at the pixel location. Because the multistatic synthetic aperture data is utilized in this approach, the estimated time delays can be injected back into the multistatic data to correct the beamforming process on both transmit and receive. By iterating this operation, the aberration is compensated, leading to cyst contrast improvement from 30 to 70% in vitro and around 10% in vivo. Notably, however, the locally-estimated phase screen is often distinct from and unlinked to the surrounding phase screens due to the use of a zero-error reference element (see Section 4.2, leading to rapidly changing brightness in the local speckle).

**Fig. 8:**
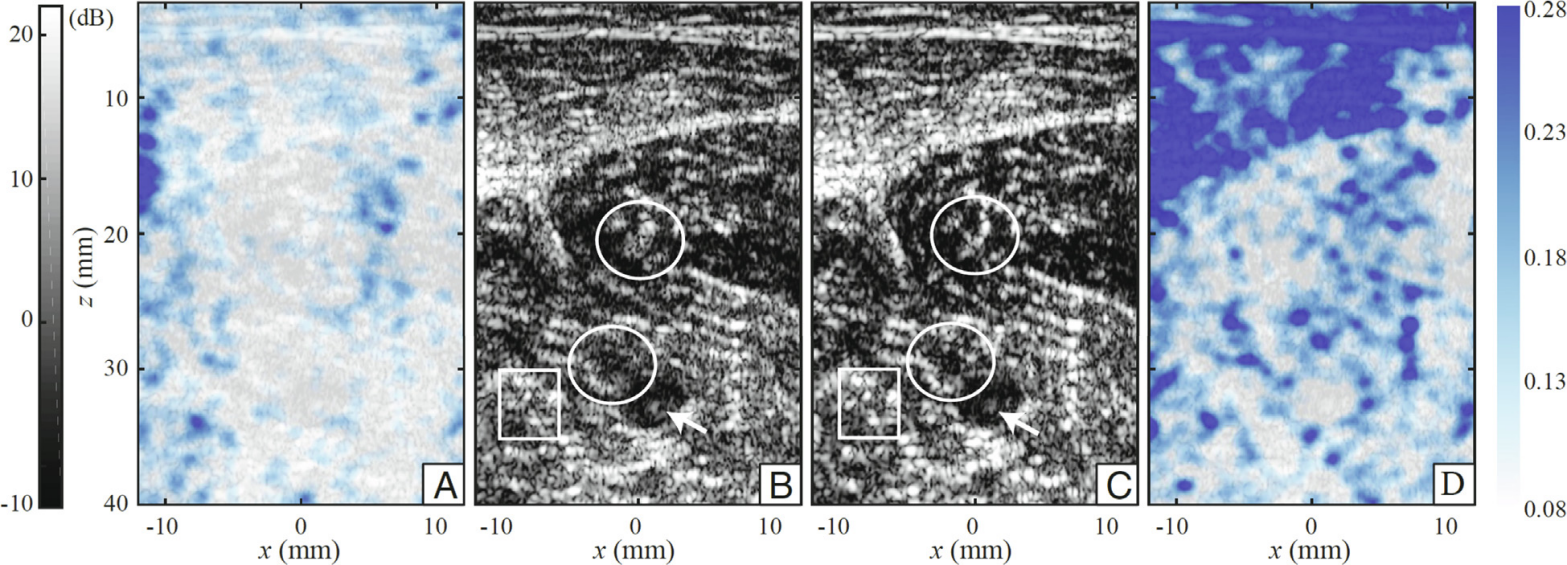
Aberration correction of in vivo imaging with the distortion matrix method of Lambert et al. [116]. Before correction, (A) the Strehl ratio map and (B) the confocal image exhibit the consequences of sample-induced distortion. (C) The corrected confocal image has improved contrast and resolution, as evidenced by the corresponding Strehl ratio map in D. Rectangular and circular areas in B and C highlight areas for comparison of images before an after correction. The Strehl ratio ranges from zero for a completely degraded focal spot to one for a perfect focusing. Reproduced from [116] (Fig. 6) with permission from The National Academy of Sciences.

Another spatially-varying phase screen correction method, based on determination of the distortion matrix, provides a full-field distributed aberration correction using the same principle of focusing ultrasound data that is backscattered by an aberrating medium [116]. The reflection matrix is built from the beamformed data for each plane wave and transducer element. Projecting this matrix into the far-field allows for the estimation and removal of the reverberation components of the aberration. Then, the wavefronts are corrected to align signals. The anti-diagonal terms of the remaining reflection matrix are the responsible for the aberration, and contain a certain amount of correlation due to the isoplanatism. In fact, within an isoplanatic patch, because the aberration is homogeneous, the distortion will also be homogeneous. For each patch, a time-reversal analysis is conducted to retrieve a central point-like scatterer that will exhibit the correction to apply. Besides correcting the image up to 14 dB in vitro, this approach may offer interesting biological markers in the distinction between single and multiple scattering regimes within the medium. An in vivo example of a transverse calf examination in a healthy volunteer is shown in (Fig. 8). The gain of image quality can be quantified by the Strehl ratio, which is defined as the ratio of the peak intensity of the imaging system point spread function with aberration to that without. The Strehl ratio ranges from zero for a completely degraded focal spot to one for a perfect focusing.

Both methods described above offer interesting and novel approaches to overcome the issue of distributed aberration, although at a high computational cost that prevents real-time correction. With this aim in mind, Bendjador et al. [117], [118], [119] proposed a formalism based on angular coherence maximization [120]. Plane wave data are beamformed individually and form the ultrafast compound matrix. Its singular value decomposition (SVD) separates the spatial and angular variation, where the maximum singular value provides the corrected image in the spatial singular vector and the angular aberration law in the angular singular vector (this is similar to the eigendecomposition techniques described in Section 4.1). This operation can either be performed at once, in the case of an aberrating screen, or on sub-regions of the image corresponding to the isoplanatic patch size. The typical patch size in liver reaches a few tens of wavelengths in azimuth and, in that context, the simple formalism of the approach still allows ultrafast imaging rates. In terms of contrast, gains of 10 dB in vitro and 6 dB in vivo can be achieved and the knowledge of the phase aberration law can be used for quantitative ultrasound imaging [117].

### 5.2 Sound speed estimation

As introduced previously, estimation of the sound speed distribution in tissue can be used as a basis for estimation of aberration correction. Significant research efforts have been ongoing in the last two decades to perform sound speed reconstruction using pulse-echo probes, as this has significant relevance to clinical sonography systems.

Early concepts for pulse-echo sound speed estimation involved measurements of the spatial shift of targets in the images when viewed from different directions [121], [122], time-of-flight measurements along incrementally tracked beams [123], [124], and echo tracking during trans-axial compression of tissue [125], [126]. Although many of these techniques could yield localized estimates of speed of sound in theory, these techniques typically required separate transducers or compression of tissue and were generally too coarse to yield an accurate sound speed measurement for pulse-echo imaging purposes, and were thus better suited for quantitative ultrasound measurements for disease identification. An early pulse-echo method based on a clinical linear array transducer, called the cross-beam tracking method showed promise in estimating sound speed [127], but was limited to a narrow wedge below the transducer and, like the previous methods, was too coarse for aberration correction.

#### 5.2.1 Global sound speed estimates

Earlier contributions to pulse-echo sound speed estimation included the estimation of the global sound speed. Although global estimate(s) of the sound speed does not enable full aberration correction itself, global estimate(s) can certainly aid in aberration correction through various means, such as serving as an *a priori* estimate or used directly in solving for the local distribution of sound speed (see Sections 5.2.2, 5.2.3). High-quality global sound speed estimation with pulse-echo ultrasound systems was shown by Anderson and Trahey [128], where the reflected waveform was captured by a linear transducer array and the arrival times of the waveform were fit to a parabola. The coefficients of the parabola were shown to directly provide good estimates of the global sound speed, even from diffuse scattering media. Using similar theory to Anderson and Trahey’s method, Pereira et al. [129] showed that the global sound speed could be found by fitting measured differential time delays profiles from backscattered signals to a model of differential time delays profiles between subsequent hyperbolic functions. Another form of global sound speed estimation utilized the registration of ultrasound images obtained from different steering directions [130]. Although this method can only provide a global estimate for sound speed, the method is highly related to subsequent methodologies on sound speed estimation using tomographic models (see Section 5.2.3).

A number of more recent techniques have applied maximization of a quality metric as a function of sound speed [131], [132], [133], [134]. In each of these cases, the quality metric (e.g. phase variance, coherence-based metric, image sharpness, etc.) was computed as a function of beamforming sound speed, and the sound speed that maximized the quality metric was identified as the global sound speed of the medium.

#### 5.2.2 Layered sound speed estimates

One approach to local estimates of sound speed is to assume a layered model of tissue. The layered model of tissue makes estimation of the localized sound speed estimation problem more tractable for pulse-echo ultrasound, however this assumption is only appropriate for some (but not all) clinical imaging scenarios.

Anderson et al. [135] proposed an adaptation to his prior method for direct estimation of global sound speed [128] wherein the interval sound speed was computed between global sound speed measurements at depth. The interval method yielded substantial errors at the sound speed transitions, even when the media consisted of discrete layers of sound speed. Also building on Anderson and Trahey’s approach, Jakovljevic et al. [136] proposed a sound speed reconstruction model for layered media based on differentiation of axial average sound speed profiles. Although similar in form to the interval sound speed method by Anderson et al. [135], the approach improved upon the estimation of local sound speed by solving a system of equations using gradient descent. Regularization of the estimates from Anderson and Trahey’s [128] technique was used to smooth noisy global average estimates and improve the convergence of the solution. Another key difference of this approach was the use of the multistatic synthetic aperture data to yield high correlations (for time delay estimation) at all pixel locations. However, this approach was computationally complex and included bias related to an *a priori* assumption of the beamforming sound speed to focus data. To alleviate this issue, Ali et al. [137] utilized a coherence-maximization approach [132] to estimate global sound speed and applied a similar solution to compute the local sound speed distribution. A similar approach to Ali et al. [137] was used in Bendjador et al. [117] to compute local sound speed estimates with an SVD beamformer using angled plane wave transmissions.

#### 5.2.3 Spatially-resolved sound speed estimates from tomographic models

Another approach to localized sound speed estimation, first introduced by Jaeger et al. [138] and called computed tomography in echo mode (CUTE), is to use reflection-tomographic approaches to reconstruct sound speed from pulse-echo ultrasound. In this approach, plane wave transmits are generated at different angles and beamformed with the full receive aperture. Non-uniformities in sound speed result in phase shifts of the receive beamformed signals between different plane wave transmit directions. Integration of these phase shifts along straight ray paths through the medium allow for estimation of the local sound speed. Importantly, in a subsequent contribution [139], receive beamforming was adjusted with respect to transmit beamforming to achieve common mid-point angles (similar to the common mid-point gather concept), which improved correlation and reduced phase estimation bias due to rotation of the point-spread function. Rau et al. [140] extended this approach to diverging wave source excitations. For phantoms with focal sound-speed heterogeneities, the use of diverging wave sources reduced diffraction artifacts with respect to plane waves, thus better complying to a ray tracing model for sound speed reconstruction and resulting in improvements in accuracy and contrast-to-noise ratio.

Beuret et al. [144] proposed a non-linear iterative operator to account for refraction effects in pulse-echo computed tomography reconstruction. In another contribution [145], the impact of different methods to estimate time delays from ultrasound channel data on sound speed reconstruction was compared, finding best results with block matching with normalized cross-correlation. Improvements in phase tracking were also achieved by correcting for dynamic range artifacts due to varying speckle echogenicity [146]. While these tomographic approaches based on difference phase shifts are effective in measuring lateral variations in the sound speed, the limited-angle nature of the problem inherently limits its ability to measure global and axial variations in layered media [147]. In addition, an initial assumption of sound speed to generate beamformed data is necessary, which introduces bias in scatterer position registration [148]. An optimization approach has been recently proposed to iteratively adjust the beamforming sound speed to reduce bias in the obtained phase estimates [149].

#### 5.2.4 Regularization and geometry considerations

Sound speed reconstruction with pulse-echo probes has been initially described as a matrix equation based on the Fourier transformation of the spatial phase shift distributions. Under some simplifications, this equation can be solved directly by multiplication with a pre-computed pseudo-inverse matrix, which can be exploited to provide real time reconstructions [138], [150]. However, each spectral component combines phase information at all spatial locations. As such, matrix-based reconstructions cannot easily adapt to missing or low-quality phase estimates. Iterative spatial domain reconstruction approaches, where ray trajectories are discretized into a spatial grid for each phase measure, can be used to overcome this limitation at the expense of reconstruction time [139], [143], [147]. Additionally, phase estimation quality measures (e.g. block-matched regression coefficients) can then be directly incorporated into the reconstruction by weighing or filtering out phase estimates at specific locations [143].

Due to the limited-angle nature of pulse-echo sound speed imaging, determining the sound speed from the phase aberration estimates is a poorly posed inverse problem, as a given aberration profile is compatible with multiple sound speed maps. Following the projection-slice theorem, we expect that measurements of ultrasound paths over an angular range to correspond to an angular range of the same size in k-space. Because the ultrasound probe is fixed at one position, the angular range is small, and thus a large amount of k-space cannot be sampled. These spatial frequencies must be inferred via regularization. Selecting a single sound speed map requires regularization or some form of prior knowledge. However, this always introduces some form of bias, potentially compromising quantitative accuracy. Recent approaches have included sparsity regularization of error terms and/or smoothness regularization of gradients across adjacent pixels. The chosen regularization norm has a significant impact in sound speed reconstruction. Tikhonov (i.e. $\ell_{2}$) regularization has shown good results for reconstructing sound speed in layered media [147], [139], [137], [151]. These types of regularization achieve closed-form solutions with a pseudo-inverse matrix. As a result, inverse problem conditioning can be conveniently assessed via eigenvalue analysis [148].

Alternatively, total variation regularization based on the $\ell_{1}$ norm has been shown to be effective in reconstructing sound speed images with sharp boundaries in focal lesion geometries [140], [143]; these boundary regions are smoothed with $\ell_{2}$ regularization. These methods have shown clinical value in quantifying sound speed increments in breast lesions [152]. Additionally these methods are robust to outliers, which are common in the case of cycle skipping errors. However, these methods are nonlinear and thus eigenvalue analysis cannot be performed, require iterative reconstruction, and are biased towards piecewise constant reconstructions. Anisotropically-weighted $\ell_{1}$ regularization has been shown to improve lesion delineation. In these methods, regularization strength varies with angle according to the available ray paths at each angle [143]. Hybrid regularization approaches may prove effective to reconstruct sound speed in arbitrary tissue geometries, which can be adjusted with additional regularization parameters [153] or adjusted with machine learning, such as a variational network architecture [154], [155]. Prior geometrical information can also be incorporated into the regularization, for instance, using B-mode images as a reference and a Bayesian framework for calculation [156], which increases reconstruction stability against phase noise.

#### 5.2.5 Distributed aberration correction techniques from estimated sound speed

**Fig. 9:**
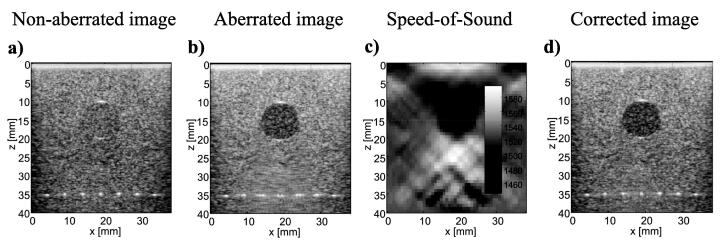
Full correction for spatially distributed speed-of-sound based on measuring aberration delays via transmit beam steering (Jaeger et al. [141]). a) Non-aberrated B-mode image of an agar phantom having a homogeneous speed-of-sound. b) Aberrated image with a second phantom containing a circular inclusion with lower speed of sound. As a result of aberration, the blurring of both point targets and diffuse echoes below the inclusion can be seeen. c) CUTE-image of speed-of-sound distribution. d) B-mode US image after aberration correction. The image shows a uniform speckle pattern with no blurring. All B-mode images were calculated by coherent averaging of plane-waves compounding from and plotted with a 60 dB dynamic range. Reproduced from [141] (Fig.3a, Fig. 3d, Fig. 4a, Fig. 4c) under License CC BY 3.0.

Recent developments in pulse-echo sound speed reconstruction in soft tissues from diffuse scattering signals have further led to exploration of how these sound speed maps can be applied for distributed aberration correction. Jaeger et al. [141] utilized sound speed to obtain an initial first order time-shift in the echo signals and then performed aberration correction based on the localized time shift estimates for different plane wave transmission angles. In this approach, point target resolution at multiple depths improved in the aberration-corrected images, and speckle texture of angular compounded images was better preserved once aberration correction was applied. By taking into account sound speed in the initial time shift, the echo positions were also properly localized (Fig. 9).

**Fig. 10:**
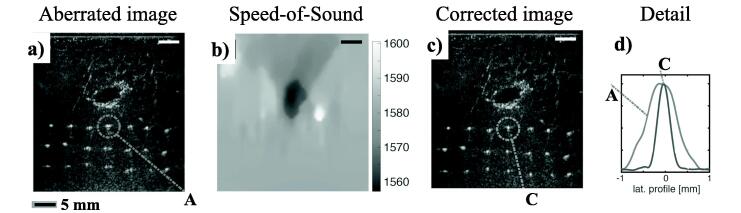
Ultrasound aberration correction based on local speed of sound (SoS) estimation (Rau et al. 2019 [142]). Results are shown for ex vivo porcine skeletal muscle with a hypoechoic gelatin inclusion and a grid of point scatterers. a) B-mode image beamformed with a global 1575 m/s SoS. b) 2D SoS reconstruction using a multiple angled plane waves and a spatial-domain reconstruction approach based on [143]. The gelatin demonstrates a considerably lower SoS compared to the surrounding muscle tissue. c) B-mode image beamformed with the local SoS-adaptive method, showing improved lateral resolution with respect to a). d) The local SoS-adaptive method leads to a narrower Gaussian shaped point-spread-function envelope in the lateral axis for the point scatterers (linear scale). All B-mode images are plotted with a 60 dB dynamic range. Reproduced from [142] (Fig. 3b-e) under License Number 5450371328909 and the terms and conditions provided by IEEE and Copyright Clearance Center.

**Fig. 11:**
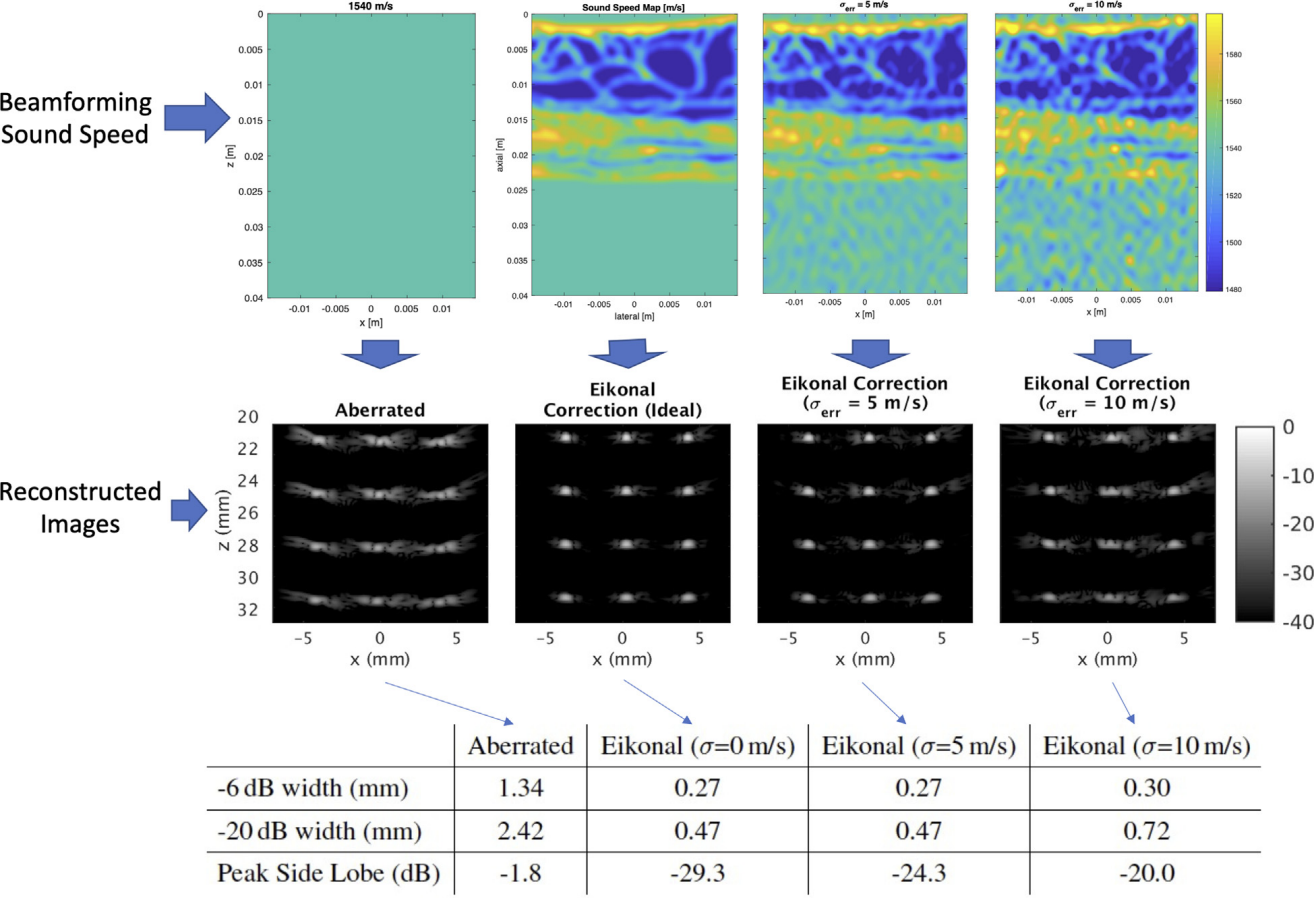
Impact of sound speed estimation errors on the focusing of point targets. The eikonal equation is used to calculate the focusing delays for each imaging point based on a given sound speed profile for the medium. For each B-mode image, the peak sidelobe lobe level in decibels (dB) and the point target resolution in terms of the average 6 and 20 dB widths of the point targets is provided. The first column of images shows the reconstructed image when the sound speed in the medium is assumed to be a uniform constant 1540 m/s throughout the medium. The second column of images shows the result when the exact ground-truth speed of sound is used to calculate focusing delays using the eikonal equation. The following two columns show the degradation in the sound speed reconstruction as errors are introduced into the sound speed profile. The root-mean-square sound speed error ($\sigma_{\mathrm{err}}$) introduce into the sound speed profile were 5 and 10 m/s. Reproduced from [161] (Fig. 4, Table 2) under license number 5450061500215 and the terms and conditions provided by IEEE and Copyright Clearance Center.

**Fig. 12:**
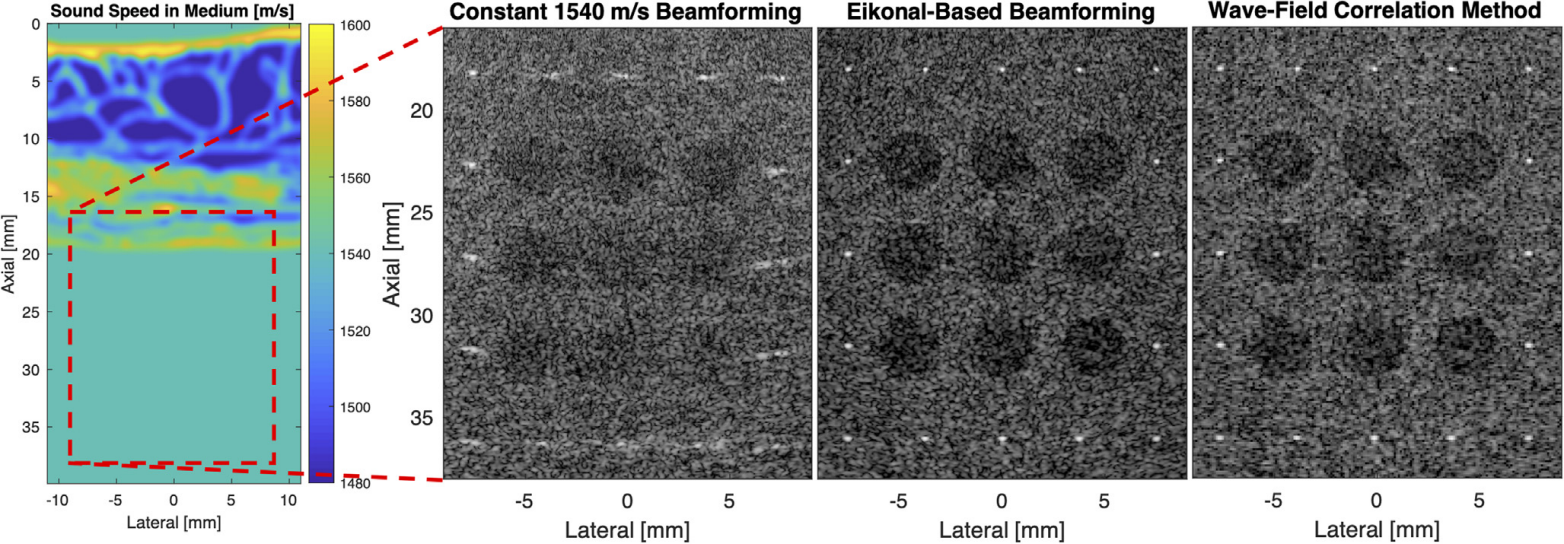
Distributed aberration correction techniques in a medium with a known heterogeneous sound speed distribution. The peak side-lobe level for point targets at 36 mm depth is 10.1 dB when beamforming at 1540 m/s, 19.1 dB when beamforming using eikonal-based travel times, and 21.3 dB when using the wave-field correlation method. The lesion contrast for the bottom row of lesions is 1.75 dB when beamforming at 1540 m/s, 6.03 dB when beamforming using eikonal-based travel times, and 6.10 dB when using the wave-field correlation method. Each image is shown at a 60 dB dynamic range.

Rau et al. [142] performed aberration correction using computation of the time delays using a straight ray model through the estimated local sound speed distribution. Significant improvement of image resolution and scatter localization in both axial and lateral directions was observed by correcting for local sound speed compared to aberration correction based on a global sound speed estimate (Fig. 10). Similarly, Ali et al. [84] performed aberration correction based on direct computation of the arrival times using sound speed estimates from a tomographic sound speed estimator for layered media. In this approach, arrival times were determined by application of the eikonal equation, thereby accounting for refracted paths. This approach showed improved point target resolution and reduced side lobes, with the aberration correction being robust against typical uncertainties in sound-speed estimation (Fig. 11). In addition to corrections based on time-of-flight, a correction technique based on the correlation of the transmitted and back-propagated receive pressure field (such as in Fig. 5) was used to correct for diffraction-related perturbations in the transmitted waveform, and showed quantitative improvements with respect to the time-of-flight approach (Fig. 12).

In transcranial ultrasound imaging, Mozaffarzadeh et al. [157] corrected phase aberration by using sound speed and geometric estimates of the skull. Although the method for sound speed estimation here only provides a single quantitative sound speed estimate of the skull, application of this sound speed to a geometrical representation of the skull allowed for ray tracing approaches (similar to Rau et al. and Ali et al.) to be applied to compute the appropriate time delays for aberration correction.

Current results on distributed aberration correction based on sound speed estimates show visual improvements in image quality with respect to non-compensated images, allowing better delineation of imaging targets through aberrating layers. Current experimental results show that the impact of these approaches has been particularly significant for strong aberrating superficial layers, such as skull or abdominal tissue layers. However, the use of distributed aberration correction based on sound speed requires more detailed validation and analysis in order to understand the spatial resolution and accuracy required of the sound speed images for sufficiently accurate aberration correction. The general principle is that sound speed estimation is only as accurate as our ability to measure aberration because the aberration itself is used to reconstruct sound speed [84]. For example, less phase aberration occurs at lower imaging frequencies, but this also means that the sound speed estimate will be less accurate because of a reduced sensitivity to phase shifts at those lower frequencies [137], [158]. The corrective ability and required accuracy of the sound speed map for aberration correction will ultimately depend on the imaging scenario and remains an active topic of research. Side-by-side comparison of distributed aberration correction with prior methods, such as those, based on phase screen models, would contribute to the understanding of performance and limitations of distributed techniques.

Thus, there is great opportunity in phase aberration correction for open source datasets and code to enable the benchmarking and comparison of phase aberration correction on standardized complex anatomical geometries. To the knowledge of the authors, there is not yet a publicly available database of complex geometries for the purpose of benchmarking aberration correction algorithms. Furthermore, it is to the knowledge of the authors, not yet common practice to make publicly available codebases of aberration correction algorithms for the purpose of baseline comparisons with new work. Some recent work that does make both code and data available publicly include Ali et al. [84]. However, this work only demonstrates a sound speed-driven approach to aberration correction with only a few sample datasets. The literature covers several different models and aberration correction techniques for which there are no open source codes and can be difficult to reproduce otherwise. Additionally, there are very few publicly available datasets with raw channel data that can be used to show images pre- and post- correction. To address this issue, the research community needs to promote the rediscovery and understanding of these other (potentially older) aberration correction techniques by making them open source and providing raw channel data as examples. Many journals have begun to promote tutorial papers to incentivize this process. Examples of open source ultrasound imaging initiatives with datasets outside of aberration correction include PICMUS [159] and CUBDL [160]. This type collective effort would greatly benefit the field of aberration correction. Further, highly public and available code-bases and datasets would enable the comparison of new methodologies of phase aberration correction and sound speed estimation with existing ones. As part of this future, guidelines and gold standards for channel data format and reconstruction could be incorporated by conference planning committees and journal editorial boards. It would be beneficial to the field, for similar challenges and datasets be performed on the task of sound speed imaging.

Lastly, current distributed aberration correction methods based on sound speed estimates have only been explored with 1-D transducer arrays. As shown by early research in aberration correction, 1.75-D and 2-D arrays significantly improved aberration correction, highlighting the need to extend sound speed estimation techniques to 3-D estimates. Acceleration of sound speed reconstruction approaches is a key element for the clinical deployment of these methods.

#### 5.2.6 Distributed aberration correction by neural network models

Recently, interest has been growing around data-driven methods to work towards the correction of phase aberration using methods from deep learning. These methods span from the estimation of the phase aberration itself or inhomogeneous sound speed fields to direct image correction and signal filtering. Estimated sound speed fields can be directly applied to increase image quality via the methods described in Section 5.2.5 and have the advantage of being highly interpretable, but are challenged by the lack of labeled training data for supervised learning methods.

Feigin et al. [162] proposed a method using an encoder-decoder neural network architecture to run supervised training on a simulated dataset of radio-frequency (RF) data to perform a spatial estimation of sound speed distributions. The input data for this approach was three plane-wave transmits from three 64 element sub-apertures of a 128 element array. This method was unique in that it used a neural network to map temporal RF data to the spatial domain in the form of an estimated sound speed distribution. The value of the sound speed was directly regressed by the neural network. The method was trained on pairs of RF data and sound speed maps of simulated in silico phantoms of randomly positioned ellipses of varying acoustic properties using the k-Wave simulation toolbox [163]. The method was implemented at high frame rates (150 fps) and identified sound speed variations in experimental phantoms, and initial results in in vivo muscle delineation. Jush et al. [164] proposed the use of a multi-input network architecture with two input branches that are passed in-phase and quadrature (IQ) channel data. The IQ channel data is generated from a single plane-wave transmission with a steering angle of zero degrees. Myeong-Gee et al. [165] proposed sound speed reconstruction from phase difference maps, with a process guided by manually segmented features in B-mode, to provide contrast and accuracy improvements with respect to end-to-end sound speed learning from radiofrequency (RF) data, showing a more robust behavior against noise in the pulse-echo data and satisfactory quantification of ex vivo and in vivo focal inclusions. In simulation studies, Heller and Schmitz [166] utilized a coherence factor as an input feature to a neural network to provide viable sound speed reconstruction that accounted for spatial positioning error of the sampled data due to heterogeneous sound speed.

In more direct applications for aberration correction, Kim et al. [167] proposed a cascaded neural network architecture for end-to-end calculation of the aberrating delay matrix for receive beamforming. In this approach, a sound speed map is first estimated from steered plane-wave transmissions as a first stage, and a second network generates a delay map for all image pixels and transducer elements. Training data was generated from full-wave simulations, where Dijkstra’s algorithm was applied to find the fastest path for reference delay matrix computation. In another study, Sharifzadeh et al. [168] estimated aberrator profiles directly from the B-mode images, using the Field II simulation package to generate training data, and applying delays to the transducer elements in both transmit and receive sides to simulate controlled aberration profiles. In simulations, the method outperformed nearest-neighbor cross-correlation [20], although as discussed in previous sections, this network model assumes a zero-mean near-field phase screen, which will limit its generalization to in vivo aberration.

In order to circumvent the requirement for labeled training data for the training of neural networks, Kahn et al. [169] proposed a self supervised method for phase-aberration correction based on the SVD beamformer [118]. In this work, a neural network was trained on the mapping between standard delay-and-sum image and the SVD-beamformed image to produce an image that has been filtered of aberration correction. Experimental results with tissue-mimicking phantoms and in vivo scans showed both visual and quantitative improvement of image quality, specially at larger imaging depths.

Although current progress in aberration correction based on neural network models is promising, further investigation is necessary to bring these methods to clinical practice. Currently, most approaches rely on simulation data for training and validation. Due to the automatic learning of features, it is not clear whether these results will generalize well to experimental and in vivo scenarios. For advancing the field, larger scale experimental validation studies are necessary. Reproducibility and diagnostic accuracy of aberration corrected images with data-driven methods also needs clinical evaluation.

### 5.3 Real-time distributed aberration correction

Ultimately, clinical deployment of distributed aberration correction techniques based on sound speed estimates and neural networks models require real-time implementation. Several approaches have been proposed to achieve fast estimations necessary for real-time aberration correction. An accelerated version of the CUTE algorithm for sound speed estimation has been proposed [150] by introducing an interpolation approximation in the integration ray paths. Sound speed estimation based on neural network approaches in graphics processing units (GPUs) has also been shown to achieve real-time frame rates [154], [162]. As for acceleration of aberration correction with full-wave methods, Schwab et al. [81] proposed an accelerated back-propagation method based on a paraxial approximation of the wave equation, which accounted for refraction and neglected unwanted back-reflections.

### 5.4 Clinical validation of aberration correction methods

Although much work has been performed in recent years to model aberration and develop approaches for aberration compensation, there is need of validation studies to show the benefits of aberration correction in clinical practice. For most of the works discussed in this review, validation of aberration correction approaches is performed with well-controlled simulation and phantom experiments. Clinical validation requires real-time implementation of aberration correction algorithms in clinical ultrasound systems, followed by definition of metrics that can objectively evaluate image improvement in in vivo scenarios. In terms of evaluating improvements in diagnostic quality, a possibility is to involve experienced image readers to blindly assess the improvement of aberration-corrected images.

**Fig. 13:**
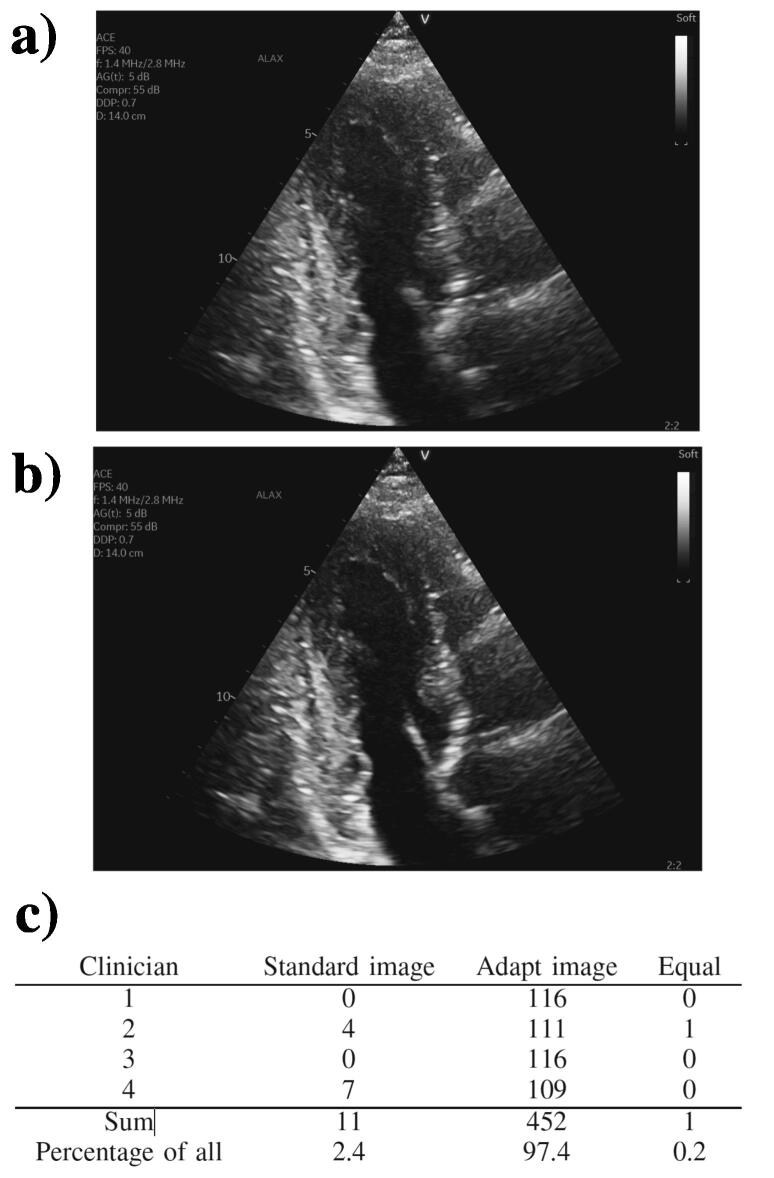
Aberration correction in 2D echocardiography (Måsøy et al. 2022 [170]). a) and b) shows apical long axis (ALAX) views from a patient with standard imaging (a) and after aberration correction (b). After aberration correction, the cardiac structures, including endocardiac borders and valves, appear thinner and more clearly defined. In the example, the mitral valve leaflets and endocardial borders become very clear after aberration correction. c) Shows a side-by-side clinical comparison of standard imaging and aberration corrected images (Adapt). The two last rows summarize the results as the accumulated sum for each column, and the percentage of each category with respect to all evaluated cineloops. Reproduced from [170] (Fig.5, Table 1) under License CC BY 4.0.

Early work of Rigby et al. [53] showed aberration correction in abdominal imaging in a population of 13 healthy males using a multi-row abdominal transducer. Great care was taken in this system to address many of the aforementioned challenges (Section 4.1). The standard deviation of the time-delay errors was 27 ns, with maximum time-delay errors below 100 ns in magnitude. Small diagnostic quality improvements could be assessed in the corrected images, such as improved visibility and contrast of known abdominal structures such as the adipose layer surrounding the renal capsule and the lower border of spleen, A reduction of clutter in blood vessels was observed with contrast improved by 1.1–1.3 dB and brightness of liver tissue increased by 1.2–2.8 dB. Dahl et al. [62] used a real-time adaptive imaging system to update the aberration profile at multiple beam locations for 1-D arrays at approximately 2 frames per second (fps) and for 1.75-D arrays at up to 0.81 fps. Using this system, they performed ultrasonic breast exams for 14 patients undergoing mammographic screening. They found a reduction of 41.6% on the width of “point-like targets” such as microcalcifications, calcifications in vessels and other tissue structures, as well as an increase in contrast-to-noise ratio for cystic lesions from 1.23 dB to 1.35 dB. In a recent echocardiography study by Måsøy et al. [170], a 2-D array was used with a modified beamsum algorithm on a software beamformer to achieve real-time imaging with aberration correction at frame rates greater than 150 frames per second; the most successful implementation of real-time aberration correction to-date. 452 images were evaluated by 4 clinicians before and after aberration correction with this system. A qualitative improvement in the images was consistently assessed in 97.4% of the images after aberration correction (Fig. 13). The global image coherence showed a median increase of 62% after correction. Cardiologists quantified a median global gain difference estimate of 1.2 dB between aberration-corrected and non-corrected images.

## 6 Conclusion

Although the deleterious effects of aberration have been recognized as early as the 1960’s, aberration correction has remained a relevant and challenging problem for ultrasound imaging. The near field phase screen model and its associated correction techniques are reasonable approaches to aberration correction and are the most amenable to real-time implementation, especially in imaging applications where a fat layer is expected near the transducer and models an infinite isoplanatic patch. However, more complex models and correction techniques are needed when the fat layers are large or the tissue is heterogeneously distributed. These techniques, while more sophisticated than the near field phase screen correction techniques, are more challenging to implement in real-time. However, with advances in modeling and computing power, the ability to correct for distributed aberration in real-time has continuously improved.

The most recent advances in aberration correction have involved sounds speed estimation to account for refraction, and potentially diffraction, while also maintaining correct spatial location of targets. New tools, including multidimensional transducer arrays and neural network estimation, are becoming more readily available and present powerful additions to the aberration correction field. The modeling, real-time implementation, and correction of aberration has improved greatly since the early days of aberration correction, and it is expected that this trend will continue with further improvements to be expected in the decades to come.

## Declaration of Competing Interest

The authors declare that they have no known competing financial interests or personal relationships that could have appeared to influence the work reported in this paper.
